# Does binge-eating matter for glycemic control in type 2 diabetes patients?

**DOI:** 10.1186/s40337-019-0260-4

**Published:** 2019-09-06

**Authors:** Marcelo Papelbaum, Rodrigo de Oliveira Moreira, Walmir Ferreira Coutinho, Rosane Kupfer, Silvia Freitas, Ronir Raggio Luz, José Carlos Appolinario

**Affiliations:** 1 State Institute Diabetes and Endocrinology, Rio de Janeiro, Brazil; 2 Group of Obesity and Eating Disorders, Institute of Psychiatry of the Federal University of Rio de Janeiro and State Institute Diabetes and Endocrinology, Avenida Ataulfo de Paiva 204, 707, Leblon, Rio de Janeiro, 22440-033 Brazil; 3 Institute for Studies in Public Health, Federal University of Rio de Janeiro, Rio de Janeiro, Brazil

**Keywords:** Binge eating disorder, Diabetes mellitus, Glycemic control

## Abstract

**Background:**

Eating behavior is an important aspect related to type 2 diabetes mellitus (T2DM) treatment and may have an impact on glycemic control. Previous reports showed elevated prevalence of eating disordered behaviors, especially binge eating disorder in clinical samples of type 2 diabetes patients. However, results regarding the impact of an eating disorder on the glycemic and clinical control of T2DM is inconsistent. The purpose of this study was to assess the impact of a comorbid eating disorder on glycemic control (GC) in a group of patients with T2DM.

**Methods:**

Eating behaviors of 70 consecutive patients with T2DM were assessed using a Structured Clinical Interview for DSM-IV and the Binge Eating Scale. The GC was examined with fasting blood glucose (FBG) and glycated hemoglobin (A1c) levels. In addition, secondary clinical variables were assessed, including body mass index (BMI) and lipids. Chi-square and Student’s T tests were used to compare clinical and psychopathological characteristics of patients with and without an ED. In order to evaluate the relationship between GC and eating disorder (ED) a linear regression analysis was performed, controlling for BMI. A significance level of 5% was adopted.

**Results:**

Seventy-seven percent of the sample (n = 54) were female and 50% were obese. Fourteen patients exhibited an ED, mostly binge eating disorder (BED). In a regression analysis, both FBG (beta coefficient = 47.4 (22.3); p = 0.037) and A1c (beta coefficient = 1.12 (0.57); p = 0.05) were predicted by the presence of an ED. However, the presence of an ED lost its impact on glycemic control outcomes after the addition of the BMI in the models.

**Conclusions:**

Eating psychopathology is frequently observed in patients with T2DM. Among individuals with T2DM, co-morbid ED is associated with a poorer glycemic control in the presence of a higher BMI. The presence of an eating disordered behavior in patients with T2DM seems to have clinical relevance in the usual care of patients with diabetes. Therefore, we recommend eating psychopathology should be routinely assessed in T2DM patients.

## Plain English summary

Eating behavior is an important aspect related to diabetes treatment. Indeed, previous studies had already observed higher rates of eating disordered behaviors, specifically binge eating disorder, in type 2 diabetes mellitus (T2DM) patients. Whether the presence of eating psychopathology has a negative impact on glycemic control of T2DM patients is a subject of controversy. Our study evaluated the relationship between eating disorder (ED) and glycemic control (and other metabolic outcomes) in patients with type 2 diabetes. We found that patients who exhibited binge eating-related eating psychopathology had worse glycemic levels than those without an ED in the presence of a higher BMI. In the usual care of people with type 2 diabetes we therefore recommend routine screening for eating disorder symptoms.

## Background

Binge-eating related psychopathology has been associated with increased risk for a variety of clinical comorbidities, not necessarily associated with overweight and obesity [1]. For instance, some reports showed binge-eating behaviors, especially binge eating disorder (BED), to be associated with increased risk of hypertension, gastrointestinal disorders and fibromyalgia [2]. In this respect, the comorbidity between type 2 diabetes mellitus (T2DM) and BED has been consistently found in prevalence studies [2, 3].

Eating behavior in T2DM might be influenced by several factors. Firstly, both eating psychopathology and type 2 diabetes could be associated with weight [4]. In addition, a restricted diet, with limited consumption of carbohydrates, is commonly prescribed as nutritional strategy for glycemic control in diabetes [5]. Lastly, binge-eating behavior might be a consequence of hypoglycemia, although it is less frequently seen in type 2 when compared to type 1 diabetes mellitus [6]. Because of the importance of eating behavior in the metabolic control of diabetes, several studies have investigated the epidemiology of the association between T2DM and eating psychopathology [7].

Herpertz et al. [8] conducted the first large multicenter study to investigate the prevalence of ED in a sample of type 1 (n = 341) and type 2 (n = 322) diabetes patients. Although the authors found similar ranges of ED prevalence between both samples, the distribution of the ED categories differed between them. In the T2DM group there was 12.6% lifetime prevalence in females (2.4% BN; 7.1% BED; 3% EDNOS) and 7.1% in males (1.3% BN; 4.5% BED; 1.3% EDNOS). In the type 1 diabetes mellitus (T1DM) group there was, for instance, 16.5% lifetime prevalence of ED, with predominance of anorexia nervosa and bulimia nervosa (BN). The most observed ED associated with type 2 diabetes was BED, with a lifetime prevalence of 5.9%. Subsequently, Kenardy et al. [9] investigated the prevalence of BED in 50 newly diagnosed T2DM patients compared to case-matched control without diabetes. A greater number of patients with type 2 diabetes (14%) reported episodes of binge eating compared to control subjects (4%). However, the diagnostic of BED did not differ between the two samples. In this way, another study also observed higher levels of regular binge eating (greater or equal twice per week over the past six months) in female (11%) and male (18%) T2DM patients [10]. Indeed, although other studies confirmed this clinical comorbidity, differences in the prevalence of ED in T2DM has varied according to the population studied, type of measurement of ED and presence of other confounding factors, especially overweight and obesity. Mannucci et al. [11], using a structured interview (Eating Disorder Examination [EDE]), found lower levels of BED in T2DM. In addition, the authors concluded that obesity, rather T2DM per se, might be a more important risk factor for eating psychopathology in T2DM. Nicolau et al. [12] also observed a positive correlation between eating psychopathology and body mass index in a sample of T2DM patients.

Although the comorbidity of an ED and T2DM has been observed across studies, the impact of this association on the clinical control of diabetes has been less consistent. Most studies investigating the clinical control of T2DM in patients with eating psychopathology focused exclusively on glycemic control, frequently using glycated hemoglobin (A1c) as the primary outcome [9, 11, 13, 14]. Two studies did not show difference on A1c between type 2 diabetes patients with and without regular binge (at least twice a week over the last 6 months) or BED, respectively [9, 14]. However, as the studies were designed specifically to investigate the prevalence of abnormal eating behavior in a diabetic population as the primary outcome, it might have lacked the power to detect a difference in the glycemic control. On the other hand, positive correlation was found between Binge Eating Scale (BES) and EDE scores and glycemic control [11, 13]. Also, only one study evaluated this impact using a longitudinal design, not showing differences on glycemic control associated with disordered eating behavior with a follow-up of 2.2 years [15].

Considering the lack of consensus regarding the impact of an eating disordered behavior on the clinical control of T2DM, the main purpose to the article was to investigate metabolic control in a clinical sample of type 2 diabetes patients with and without an ED.

## Research design

Seventy outpatients with T2DM, aged 18 to 65 years, were assessed consecutively at the Diabetes and Endocrinology State Institute of Rio de Janeiro. Diabetes was diagnosed according to the American Diabetes Association criteria [16]. Patients with T1DM, gestational diabetes, secondary diabetes due to another disease or using medication likely to affect food intake (antidepressants, anti-obesity agents) were excluded from the analysis. Patients who met the inclusion criteria were consecutively evaluated after informed consent was obtained.

## Methodology

### Clinical examination

Screened patients were asked to come to a scheduled visit. Anthropometric examinations and laboratory tests were obtained in the same day, early in the morning, to assess the metabolic control of diabetes. Weight was measured in light street clothes, without shoes, on a calibrated balance-beam scale and, subjects’ heights were measured using a stadiometer. Body mass indices (BMI) were calculated by dividing weight (kg) by height squared (m2). Glycemic control of diabetes was assessed measuring the levels of fasting blood glucose (FBG) and A1c. FBG was measured using a colorimetric enzymatic method, and A1c levels were determined using high-performance liquid chromatography (HPLC; Variant turbo, Bio-Rad, National Glycohemoglobin Standardization Program). Clinical characteristics of diabetes were collected from the medical records, including duration of diabetes, insulin and oral anti-diabetics use and the presence of microvascular complications related to diabetes (retinopathy, nephropathy and neuropathy).

### Psychiatric instruments

All subjects were interviewed by a psychiatrist (MP) trained in the use of the Structured Clinical Interview for DSM-IV, patient edition (SCID-P) [17]. This instrument was used for the diagnosis of ED. The BES was also applied to provide a measure of severity of eating psychopathology [18]. This instrument has been translated into Portuguese, presenting good reliability [19, 20].

### Data analysis

Continuous variables were expressed as means and standard deviations, and categorical data were described as absolute and relative frequencies. Patients with ED were compared to those with no eating psychopathology in terms of clinical and psychopathological characteristics using non-paired t tests to compare means of continuous variables and the Chi-square test to analyze categorical variables. In order to analyze the relationship between diabetes glycemic control and eating psychopathology, a linear regression was performed. Two different models were considered, using A1c and FBG as the main outcome (dependent variables) and eating disorder diagnosis status as an independent variable. Also, the BMI was later added in both models as an independent variable to evaluate possible confounding factor. A significance level of 5% was adopted. The statistical analysis was conducted using SPSS software.

## Results

A total of 113 diabetes outpatients were consecutively screened. After the observance of exclusion criteria, our final sample consisted of 70 T2DM individuals. All individuals who met the inclusion criteria agreed to participate in the evaluation. Our study sample consisted mostly of females (77%), married individuals (70%) and individuals who had less than nine years of schooling (71%) with a mean age was 52.9 ± 6.8 years. Mean BMI was 30.6 ± 5.2 kg/m^2^. Half of the individuals were obese (mean BMI = 34.8 ± 3.5 kg/m^2^), 22 were overweight (mean BMI = 27.9 ± 1.1 kg/m^2^) and 13 exhibited normal weight (mean BMI = 23.6 ± 1.2 kg/m^2^). The sample consisted of patients with a long illness duration (13.33 ± 7.55 years), with high rates of neuropathy (22%), retinopathy (42%) and nephropathy (52%). In addition, 51% of the patients were using insulin regularly.

The prevalence of ED was 20%. The most frequently observed condition was BED, observed in 7 of the 14 the participants with an ED. In addition, BN was diagnosed in 3 patients, and 4 individuals exhibited an eating disorder not otherwise specified (EDNOS), with subclinical BED. Because of the low number of bulimic and subclinical BED individuals, it was decided to analyze the group as a whole, with a binge eating-related ED. The rate of an ED varied according to BMI status. Specifically, normal-BMI individuals exhibited a rate of ED of 8%, contrasted with a 26% prevalence of ED in obese patients. In addition, there was a positive correlation between eating psychopathology severity (using BES scores) and BMI (r = 0.24; p = 0.04) However, patients with ED did not differ from those without an ED diagnosis regarding to the presence of insulin use (50% vs 52%; p = 0.9). In addition, the presence of a binge-eating related disorder did not have an impact on metformin mean dosage use (2007.1 ± 514.3 mg vs. 2030.3 ± 628.9 mg; *p* > 0.05).

Table 1 shows the comparison of clinical and psychopathological features between patients with and without a ED. None of those characteristics was associated with the presence of an ED. In addition, the subjects with T2DM and a co-existing ED exhibited higher levels of eating psychopathology severity, measured by BES scores, compared to those without an ED.

**Table 1:** Clinical and psychopathological comparison between type 2 diabetes patients with and without eating disorders

	T2DM	T2DM	T2DM	*p* value
	Total sample	Without eating disorder	With eating disorder	
	*n* = 70	*n* = 56	*n* = 14	
Age (years)	52.9 ± 6.8	53.2 ± 6.9	51.8 ± 6.8	0.50
Diabetes duration (years)	13.4 ± 7.5	13.6 ± 7.5	12.2 ± 8.0	0.52
Body mass index (kg/m2)	30.6 ± 5.2	30.0 ± 5.2	32.6 ± 4.8	0.09
Total cholesterol (mg/dl)	199.8 ± 44.2	199.1 ± 44.5	202.3 ± 44.5	0.81
Triglycerides (mg/dl)	131.9 ± 55.5	128.4 ± 50.3	145.0 ± 72.8	0.34
Binge eating scale	12.8 ± 10.3	9.5 ± 8.1	25.6 ± 8.1	< 0.001

Data are presented as mean (SD). *T2DM* Type 2 diabetes mellitus

In a regression analysis (Table 2), ED status predicted both FBG and A1c levels. However, the addition of the BMI in the two models had an impact on the results and, therefore, the presence of an ED lost its influence on glycemic control outcomes.

**Table 2:** Regression analysis of glycated hemoglobin ang fasting blood glucose according to the presence of an eating disorder before and after controlling for body mass index

Model	R	Unstandardized Beta Coefficients (Std. error)	*p* value
1- Dependent Variable: A1c			
ED	0.25	1.12 (0.57)	0.05
ED	0.28	0.99 (0.58)	0.09
BMI		0.05 (0.05)	0.32
2- Dependent Variable: FBG			
ED	0.26	47.4 (22.3)	0.037
ED	0.29	42.3 (22.7)	0.067
BMI		1.9 (1.8)	0.274

*A1c* Glycated hemoglobin, *BMI* Body mass index, *ED* Eating disorder, *FBG* Fast blood glucose

## Discussion

In our study, 20% of patients with T2DM had an ED, predominantly BED. Also, those with an ED had higher levels of obesity. In addition, patients with comorbid ED had a poorer glycemic control compared to those with normal eating behaviors, not related specifically to age or duration of diabetes. However, when including BMI in the regression model, the impact of eating psychopathology on A1c and FBG disappeared, showing that body weight may play a major role modulating the relationship between ED disturbances and glycemic control.

Elevated rates of eating psychopathology in type 2 diabetes individuals has been found in several studies. However, heterogeneity of results could be influenced by the clinical characteristics of the sample and the instruments used for ED diagnosis. Evaluating those who displayed regular binge eating episodes or presented with one or more positive response as per Questionnaire of Eating and Weight Pattern scoring criteria, rates of eating psychopathology found in type 2 diabetes patients were 14 and 40%, respectively [9, 13]. On the other hand, the use of a more restrictive diagnostic instrument may have led to lower estimates of ED numbers. For instance, using the EDE, Mannucci et al. [11] found low rates of BED (2.5%) in T2DM female patients with obesity. In the Look AHEAD trial [21] 14% of the 845 T2DM patients were screened positive for BED (using Eating Disorder Examination Questionnaire) or night eating syndrome (NES), using NES questionnaire. When using the EDE interview or the Night Eating Syndrome History and Inventory (NESHI) to confirm the diagnosis, only 5.2% of those were diagnosed an ED. The results of our study corroborate the high rate of eating psychopathology seen across studies. Although we also used a semi-structured interview for ED diagnosis, the longer duration of diabetes and the high mean age of the patients could have influenced the prevalence of ED found in our study, as it was higher from those studies that used more structured interviews. Indeed, Gagnon et al. [10] showed that ED-T2DM patients had a younger onset of diabetes than the type 2 diabetes subjects without ED.

Previous studies that investigated metabolic control in the comorbidity between T2DM and ED were heterogeneous and showed different results. A poorer glycemic control in T2DM exhibiting an ED was also observed in other studies. However, different from our investigation, they did not use a semi-structured interview for the diagnosis of ED. Mannucci et al. [11], evaluating eating psychopathology in 156 T2DM patients, observed a positive correlation between A1c levels and EDE scores. More recently, Meneghini et al. [13], evaluating a multiethnic sample of T2DM patients, found statistically higher levels of A1c in patients who exhibited binge-eating episodes compared to those who did not (*p* = 0.027). On the other hand, other investigations did not observe this association. Nicolau et al. [12] found similar levels of A1c and FBG in T2DM patients despite the presence of a comorbid ED. Crow et al. [22] evaluated eating psychopathology in 43 consecutive T2DM patients. Patients with type 2 diabetes who exhibited binge eating showed no differences in A1c levels compared to their non-binge eating counterparts (*p* = 0.553). Similarly, Kenardy et al. [9] investigated the binge-eating diagnosis in 215 females with T2DM. No difference was observed in mean A1c levels in T2DM patients who binged regularly compared to those who displayed no eating psychopathology. Herpertz et al. [23] also found no difference in A1c levels in TD2M patients with an ED compared to those who exhibited no eating psychopathology (*p* = 0.26). Finally, Ryan et al. [10] found similar A1c levels in type 2 diabetes patients who exhibited abnormal eating behaviors compared to those who had normal eating behaviors. These conflicting results could reflect differences in regard to sample size, duration of diabetes and assessment of an ED and its severity.

In our study, the levels of A1c and FBG were predicted according to ED occurrence. However, after controlling for BMI these associations lost statistical significance. In fact, our results are in line with previous notion that weight is a major contributor to metabolic and glycemic control in individuals with type 2 diabetes [24]. Indeed, some authors struggled to demonstrate an independent impact of eating psychopathology on glycemic control, when controlling for weight status [11]. As a matter of fact, the relationship between eating behavior and weight is so intrinsic that both variables should be accounted together when evaluating glycemic control. Nevertheless, it is not possible to rule out that diabetes-specific treatment features and the intermittent natural course of binge-eating psychopathology could also have influenced some of the results.

Generally, levels of A1c and FBG should share a similar trend when evaluating glycemic control. However, is important to note that they measure different aspects of glycemic control. The American College of Endocrinology guidelines for glycemic control states that periodic measurements of A1c levels (which represent a 2- to 3-month average blood glucose concentrations) should be made. In addition, regular measurements of FBG (which indicates acute glycemic control) should also be included [22]. However, it is not uncommon for clinicians who treat people with diabetes to find individuals in whom A1c and FBG do not match [25]. Different from previous studies, we decided to evaluate the relationship between binge-eating related psychopathology with both A1c and FBG in order to assess specific aspects of glycemic evaluation. Although it is not possible to address any interpretation with the data presented, it seems interesting to discuss that, regarding to BED patients, the presence of late evening binge eating (raising FBG) coupled with daytime food restriction (lowering A1c) could result in some discrepancy between FBG and A1c levels. Nevertheless, in order to address this hypothesis specific methodological procedures would be necessary, for instance, the use of 24-h food diaries. In addition, evaluation of glycemic variability, with continuous glucose monitoring, could help understand differences between FBG and A1c and investigate a possible role of binge eating on dysglycemia (peaks and nadirs) [26].

Although glycemic control has been the gold-standard when investigating the impact of eating psychopathology on T2DM, other diabetes-related variables could be secondarily analyzed. For instance, although Crow et al. [14] did not find worse clinical control of T2DM in patients with ED compared to those without ED, the authors observed that patients who exhibited regular binge eating episodes used a greater number of medications for diabetes management than did those who did not binge frequently. The same situation occurred in the study by Kenardy et al. [9], who observed that the percentage of T2DM patients who used insulin or more than one hypoglycemic drug was higher in those patients who binged regularly than in those who did not. In addition, those patients who binged had a lower adherence to diabetes diet and exercise recommendations. Finally, Nicolau et al. [12] observed higher triglycerides levels in T2DM patients with an ED compared to those without an eating abnormality. Differently from Kenardy et al., our study did not find an association between insulin use and ED diagnosis. Overall, our findings are consistent with the majority of previous research that did not find an association between eating psychopathology and general diabetes clinical or treatment features.

Some limitations of this study must be discussed. As this was an exploratory study, sample size calculation was not made and might have limited the power to detect some of the associations. Also, control for multiple variables was avoided due to sample size. The term ED was used to refer to binge eating-related diagnosis and, as only 3 patients were diagnosed with bulimia, analysis by ED category was not possible. In addition, it could be argued that the inclusion of 4 patients with EDNOS with subclinical BED (which would be characterized in DSM-5 as either BED or other specified feeding or eating disorder [BED type]) could have limited the severity of eating psychopathology and, subsequently, the power to detect associations. However, the sample of ED individuals seemed to reflect well the presence of eating psychopathology as BES scores were markedly high and differed between those with and without ED. Nevertheless, it is not possible to rule out that subjects with more severe ED could have been selected out, as those taking psychotropics were excluded. The use of a broader multidimensional evaluation, including food diary, general psychopathology and diabetes-specific quality of life measures could have added information regarding other aspects of the clinical control of diabetes. On the other hand, the use of a semi-structured interview for ED diagnosis, associated with a self-report instrument to assess severity of eating psychopathology should be considered one strength in methodology, especially compared to most of the studies in this field.

## Conclusions

The pool of evidence regarding the association between ED and T2DM seems to justify screening diabetic patients for abnormal eating behaviors. In addition, when obesity is present, eating psychopathology investigation is even more recommended, since it may disrupt obesity treatment and indirectly affect diabetes control.

Although the objective negative clinical impact of an ED on type 2 diabetes control is yet to be confirmed, is possible to speculate that the remission of binge episodes could play a major role in diabetes treatment. The clinical control of eating psychopathology could enhance nutritional recommendations adherence and may diminished post-prandial glycemic peaks. Nevertheless, although the spectrum of the clinical significance of the comorbidity of ED and T2DM has not been extensively studies, treatment of binge-eating related disorders could improve perception of self-efficacy of patients toward the diabetes dietary carbohydrate goals and, ultimately improved diabetes-related quality of life.

## Data Availability

The datasets used and/or analyzed during the current study are available from the corresponding author on reasonable request.

## Abbreviations

A1c: Glycated hemoglobin
ADA: American Diabetes Association
BDI: Beck depression inventory
BES: Binge Eating Scale
BMI: Body mass index
ED: Eating disorder
EDE: Eating Disorder Examination
FBG: Fasting blood glucose
GC: Glycemic control
NES: Night eating syndrome
SCID-P: Structured Clinical Interview for DSM-IV, patient edition
T2DM: Type 2 diabetes mellitus
